# Fermented broccoli stalk by-product with lactic acid bacteria ameliorates high-fat diet-induced obesity in C57BL/6 mice

**DOI:** 10.3389/fnut.2025.1670009

**Published:** 2025-10-09

**Authors:** Lihao Jiang, Qinghang Wu, Zhiyi Lu, Jianming Zhang, Dandan Zhao, Daqun Liu, Chengcheng Zhang

**Affiliations:** ^1^College of Biological and Environmental Sciences, Zhejiang Wanli University, Ningbo, China; ^2^Food Science Institute, Zhejiang Academy of Agricultural Sciences, Hangzhou, China; ^3^Ecology and Health Institute, Hangzhou Vocational & Technical College, Hangzhou, China

**Keywords:** fermentation, broccoli stem by-product, lactic acid bacteria, high-fat diet, obesity, gut microbiota

## Abstract

**Introduction:**

Lactic acid bacteria (LAB) fermentation of broccoli stem by-product (BsBP) has been shown to enhance the accumulation of bioactive isothiocyanates, thereby possessing the potential as an anti-obesity functional food. However, the obesity-alleviating effects of fermented BsBP with LAB have not been extensively studied.

**Methods:**

In this study, BsBPs were fermented by a single strain of *Lacticaseibacillus casei YC5* or *Pediococcus pentosaceus* RBHZ36, and their impact on obesity-alleviating was investigated in C57BL/6 mice subjected to a high-fat diet (HFD).

**Results and discussion:**

Fermented BsBP intervention markedly limited body weight gain and adipose coefficients, improved serum lipid profiles and glucose levels, and reduced hepatic levels of ALT, AST, and the pro-inflammatory cytokine TNF-α in these mice. Fermented BsBP with LAB exhibited more effective anti-obesity effects than fresh BsBP in HFD-fed mice. Furthermore, supplementation with fermented BsBP modulated the gut microbial imbalance induced by an HFD, enhancing both microbial diversity and richness. This intervention promoted the proliferation of health-associated bacterial genera such as Akkermansia and Bacteroides while concurrently reducing the abundance of taxa linked to obesity, including Colidextribacter, Helicobacter, and Mucispirillum. Predictive analysis of microbial function indicated that alterations in the gut microbiota were linked to enhanced energy metabolism and activation of the PPAR signaling pathway. Collectively, this study highlighted that BsBP fermented with LAB has the potential to be a functional food for obesity management.

## 1 Introduction

Lipid metabolism, a critical and intricate process in the body, can be disrupted by excessive intake of saturated fats and dietary sugars, leading to an energy imbalance and subsequent obesity (1, 2). Obesity has increasingly become a worldwide health issue, playing a significant role in the development of chronic illnesses like type 2 diabetes and high blood pressure while also exerting considerable mental and emotional strain on those affected (3, 4). Accumulated evidence suggests that, compared to pharmacological treatments and surgical interventions with notable side effects (5), dietary strategies such as probiotic-fermented vegetables may mitigate the adverse effects of treatment because of their bioactive ingredients (6), thereby suppressing weight gain and improving metabolic health (7, 8).

Broccoli stem by-product (BsBP), a discarded food after harvesting edible florets, is rich in glucosinolates (GSLs). However, GSLs do not possess biological activities (9). Our previous research indicated that GSLs in vegetables can be biotransformed into isothiocyanates (ITCs) after fermentation (10). Interestingly, the released ITC compound from GSL has multiple excellent physicochemical and functional qualities. Sulforaphane (SFN), an isothiocyanate produced from glucoraphanin, counteracts visceral fat accumulation, enlargement of adipocytes, insulin insensitivity, and oxidative lipid damage induced by a high-fat diet (HFD), primarily through the activation of Nrf2-Related Mechanism (11, 12). In addition, indole-3-carbinol (I3C), a breakdown product of glucobrassicin, exhibits promising biological activities, including the inhibition of fat synthesis, reduction of inflammation, and antioxidant properties (13, 14). These findings suggest that fermentation has emerged as a superior method for effectively enhancing the biological activity of broccoli stems, and the fermented BsBP with enhanced ITCs may be a potential functional food.

Lactic acid bacteria (LAB) play a pivotal role in GSL degradation during the fermentation of Brassica vegetables (10, 15). The use of LAB fermentation to release GSLs from plant tissues and convert them into bioactive ITCs has become a research focus (16). Tolonen et al. (17) demonstrated that LAB starter containing *Leuconostoc* (*Leu.*) *mesenteroides* and *Pediococcus* (*P.*) *dextrinicus*-induced fermentation of cabbage facilitated the degradation of glucobrassicin into I3C. Moreover, Peñas et al. (18) used *Leu. mesenteroides* and *Lactiplantibacillus plantarum* to ferment white cabbage, also showing an increase in iberin (an ITC derived from glucoiberin) and SFN content in the final product. In earlier work, we identified *Lacticaseibacillus casei* YC5 and *Pediococcus pentosaceus* RBHZ36 as effective lactic acid bacteria capable of converting glucosinolates into isothiocyanates; additionally, incorporating these strains into BsBP fermentation substantially increased the yield of ITC-derived metabolites (i.e., SFN, I3C, and ascorbigen) (19). Considering the potential beneficial effect of ITCs and probiotic LAB, we thus hypothesize that the fermented BsBP with LAB may exhibit lipid-reducing effects and be beneficial as a functional dietary intervention for managing obesity.

The objective of the current study was to evaluate the anti-obesity effects of LAB-fermented broccoli stem byproducts in C57BL/6 mice fed a HFD. Herein, *Lacticaseibacillus casei* YC5 and *Pediococcus pentosaceus* RBHZ36, both capable of efficiently converting glucosinolates into isothiocyanates, were used to ferment BsBP over a 24-h period. Subsequently, the BsBP fermented with *Lb. casei* YC5 or *P. pentosaceus* RBHZ36 was administered to HFD-induced obese mice, and their influence on obesity-specific physiological parameters and gut microbial composition was evaluated to provide a theoretical foundation for developing fermented BsBP as a functional food.

## 2 Materials and methods

### 2.1 Materials

The BsBPs used in this study were sourced from a local farm produce market in Hangzhou, China. Orlistat was acquired from Zhongshan Wanhan Pharmaceutical Co., Ltd., located in Zhongshan. Haibo Biotechnology Co., Ltd. in Qingdao High-Tech Industrial Park supplied the MRS culture medium. Biochemical assay reagents were purchased from Nanjing Jiancheng Bioengineering Institute, Nanjing. Both the HFD (containing 45% fat) and the low-fat diet (with 10% fat) were provided by Dize Biotechnology Co., Ltd. in Wuxi.

### 2.2 Fermentation procedure for BsBP

Bacterial strains of *Lb. casei* YC5 and *P. pentosaceus* RBHZ36 were first propagated in MRS broth at 37 °C for a 24-h incubation period. Following cultivation, the cultures were subjected to centrifugation at 8,000 rpm for 10 min to collect the bacterial cells. After discarding the supernatant, the resulting pellets were rinsed twice with sterile distilled water and then resuspended in sterile water to yield cell suspensions with a final concentration of approximately 10^8^ CFU/mL.

Fresh BsBPs were cut into 1 × 1 × 2 cm cuboid-shaped pieces and mixed with 4% NaCl saline water at a 1:2 ratio (w:v), and subsequently fermented using the methods we previously described (19). In brief, the BsBP were, respectively, inoculated with 0.5% (v/v) *Lb. casei* YC5 or *P. pentosaceus* RBHZ36 suspension (10^8^ CFU/mL), and fermented for 24 h at room temperature (25–30 °C) in the airtight pickle jar. After 24 h of fermentation, the pH of the fermented BsBP dropped to 4.0, indicating that the fermentations had reached maturity (10). Then, the BsBP fermented with *Lb. casei* YC5 and *P. pentosaceus* RBHZ36 were freeze-dried, respectively. Additionally, fresh broccoli stems were freeze-dried directly without fermentation to serve as a control group. The specimens were kept at −80 °C until further use. The chemical compositions of fermented BsBP were determined, as detailed in Table 1.

**Table 1:** The chemical composition of the fermented broccoli stalk by-product (BsBP).

Determination	Fresh BsBP	BsBPs fermented by RBHZ36	BsBPs fermented by YC5
Moisture (g/100 g)	92.15 ± 0.06 a	91.80 ± 0.04 b	91.72 ± 0.06 b
Ash (g/100 g)	1.08 ± 0.02 b	2.72 ± 0.01 a	2.74 ± 0.01 a
Protein (g/100 g)	1.70 ± 0.02	1.72 ± 0.04	1.73 ± 0.01
Fat (g/100 g)	0.30 ± 0.01	0.28 ± 0.01	0.28 ± 0.01
Carbohydrates (g/100 g)	0.65 ± 0.01 a	0.20 ± 0.01 c	0.34 ± 0.00 b
Total GSLs (mg/100 g)	46.96 ± 0.21 a	34.85 ± 0.16 b	31.36 ± 0.15 c
SFN (mg/100 g)	1.94 ± 0.04 b	3.54 ± 0.04 a	3.38 ± 0.10 a
I3C (μg/100 g)	7.88 ± 0.19 c	21.18 ± 0.95 b	35.89 ± 1.12 a
Ascorbigen (mg/100 g)	0.27 ± 0.01 b	1.99 ± 0.09 a	2.03 ± 0.10 a

Means with different letters within a column are significantly different (*p* < 0.05).

### 2.3 Animal experiments

Male C57BL/6 mice, aged 6 to 7 weeks and weighing approximately 21 ± 2 g, were maintained under controlled environmental conditions, including a temperature of 23 ± 2 °C, a relative humidity range of 40–70%, and a 12-h light/dark cycle. All animal handling and experimental procedures received ethical approval from the Institutional Animal Care and Use Committee of the Zhejiang Academy of Agricultural Sciences (Protocol No. 2024ZAASLA108).

**Figure 1:**
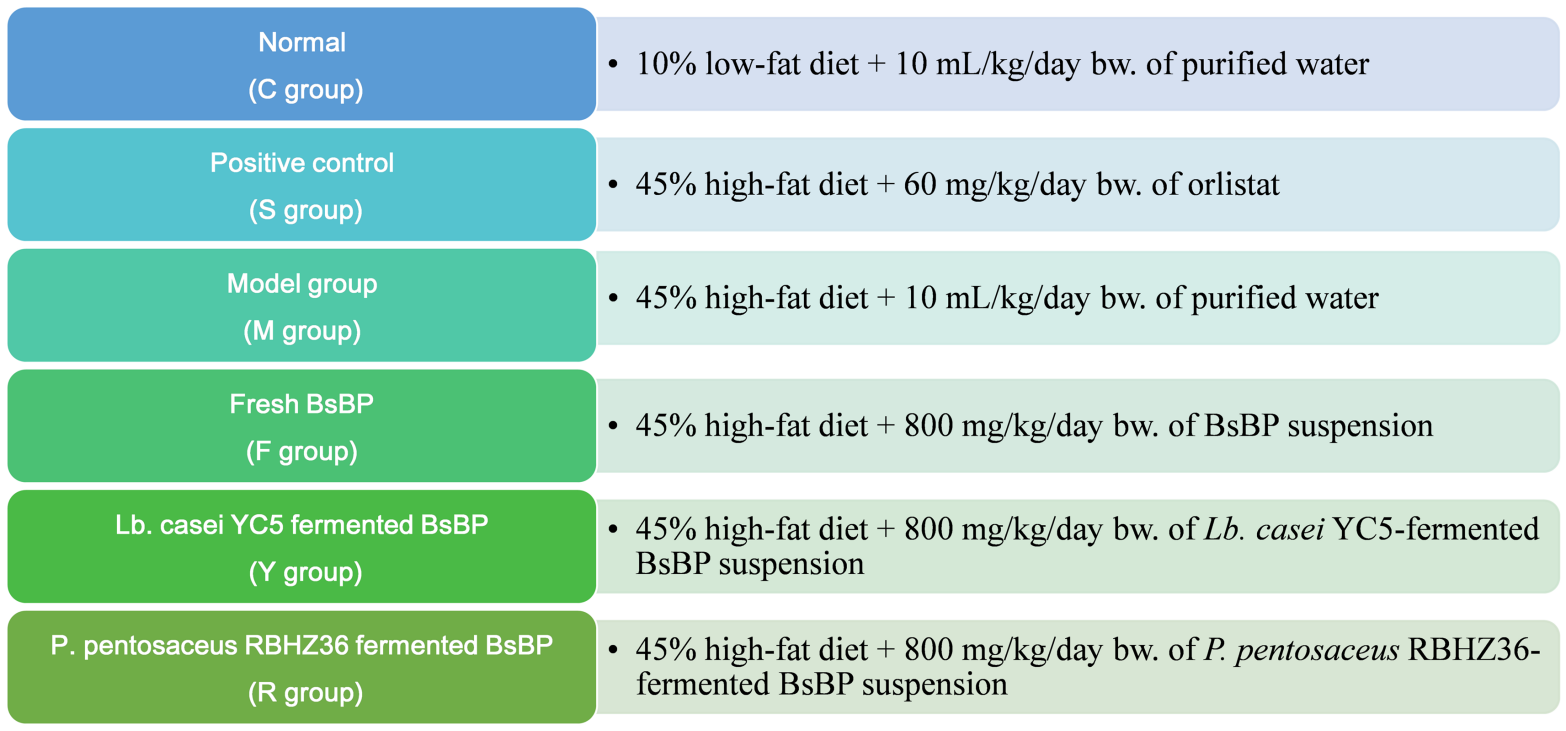
The treatment flow chart of animal experiment design. C: control group, mice fed low-fat diet; M: model group, mice fed HFD; S: positive control group, mice fed HFD and received 60 mg/kg of orlistat; F: fresh BsBP group, mice fed HFD and received 800 mg/kg of fresh BsBP; Y: *Lb. casei* YC5-fermented BsBP group, mice fed HFD and received 800 mg/kg of YC5-fermented BsBP; R: *P. pentosaceus* RBHZ36-fermented BsBP group, mice fed HFD and received 800 mg/kg of RBHZ36-fermented BsBP.

Following a seven-day acclimatization period during which mice had unrestricted access to food and water, they were assigned into six groups by simple randomization and blinding procedures, with 10 animals per group (Figure 1). The control group (C) received a 10% low-fat diet and was administered purified water (10 mL/kg body weight) daily via oral gavage. The HFD model group (M) was fed a 45% fat diet, along with the same volume of water, via oral gavage. The positive control group (S) consumed the HFD and received 60 mg/kg body weight of orlistat. Mice in the fresh BsBP group (F) were fed an HFD and administered 800 mg/kg body weight of freeze-dried broccoli stem by-product suspension. The group receiving BsBP fermented with *Lacticaseibacillus casei* YC5 (Y) was fed the HFD and treated with 800 mg/kg body weight of the YC5-fermented BsBP. Similarly, the group given *Pediococcus pentosaceus* RBHZ36-fermented BsBP (R) received the same HFD and dose. The selection of oral gavage dose of BsBP was determined through preliminary experiments demonstrating no adverse effects in mice, which was equal to 5,280 mg/day in a 60 kg adult according to dose translation formula (20).

The HFD and oral gavage intervention were conducted over a period of 12 weeks. On the day preceding the experiment’s end, the mice underwent an overnight fast, followed by orbital blood collection, and were then euthanized using cervical dislocation. Perirenal and epididymal adipose tissues were harvested and weighed, while liver and adipose tissue sections were fixed in formalin to enable subsequent histological examination.

### 2.4 Biochemical analysis

Whole blood was collected from the orbital vein and allowed to coagulate for 2 h at 4 °C. The clotted blood was then centrifuged at 3000 rpm at 4 °C for 15 min to obtain the serum. The isolated serum was aliquoted and kept at −80 °C until biochemical testing. Quantification of total cholesterol (TC), triglycerides (TG), high-density lipoprotein cholesterol (HDL-C), low-density lipoprotein cholesterol (LDL-C), alanine aminotransferase (ALT), aspartate aminotransferase (AST), and glucose levels were conducted using commercial assay kits in accordance with the manufacturer’s protocols.

Liver samples were processed by blending one part of tissue with nine parts of physiological saline to produce a 10% (w/v) homogenate using a high-speed homogenizer. The mixture was then centrifuged at 3000 rpm for 15 min, and the supernatant was retained for further evaluation. Concentrations of hepatic total cholesterol (TC), triglycerides (TG), alanine aminotransferase (ALT), and aspartate aminotransferase (AST) were determined using standard commercial kits. Moreover, liver levels of tumor necrosis factor-alpha (TNF-α) were measured using an enzyme-linked immunosorbent assay (ELISA) kit.

### 2.5 Histopathological studies

Samples of liver and adipose tissue were immersed in 10% neutral-buffered formalin for a 24-h fixation period, dehydrated with gradient ethanol, cleared with xylene, embedded in paraffin, and automatically serially cut into sections with a thickness of 5 μm. For staining, the sections were first soaked in hematoxylin staining solution and rinsed with distilled water, then differentiated in 1% hydrochloric acid ethanol and rinsed again with distilled water, and finally stained with eosin staining solution and rinsed once more with distilled water. After staining, the slides were dehydrated with gradient ethanol, cleared with xylene, dried, and sealed with neutral gum. Histological evaluation was performed under a light microscope to determine the extent of tissue damage and structural alterations.

### 2.6 Changes in gut microbiota analysis

Fecal genomic DNA was isolated using a commercial extraction kit designed for stool samples. DNA quality, including concentration, purity, and structural integrity, was confirmed using 2% agarose gel electrophoresis. The V4–V5 regions of the bacterial 16S rRNA gene were then amplified through polymerase chain reaction (PCR). The resulting PCR products were purified using the AxyPrep DNA Gel Extraction Kit. Hangzhou Lianchuan Biotechnology Co., Ltd. then performed sequencing after constructing the sequencing library. Sequence data were grouped into operational taxonomic units (OTUs) with a 97% similarity threshold using Uparse (version 11). Taxonomic identification of representative OTUs was conducted by comparison with the SILVA database, and microbial composition was analyzed across taxonomic ranks using the LC-Bio Cloud Platform.^1^

### 2.7 Statistical analysis

All statistical analyses were performed using IBM SPSS Statistics version 21, Normal distribution were assessed employing the Shapiro–Wilk test. To assess group differences, one-way analysis of variance (ANOVA) was applied with Tukey’ multiple comparisons test. Results are expressed as mean values accompanied by the standard error of the mean (SEM), with significance assigned to comparisons yielding a *p*-value below 0.05. Groups exhibiting statistically distinct outcomes are indicated using unique superscript letters. Graphical representations were generated using GraphPad Prism version 9.5.0.

## 3 Results and discussion

### 3.1 Effect of fermented BsBP on body weight of HFD-fed mice

**Figure 2:**
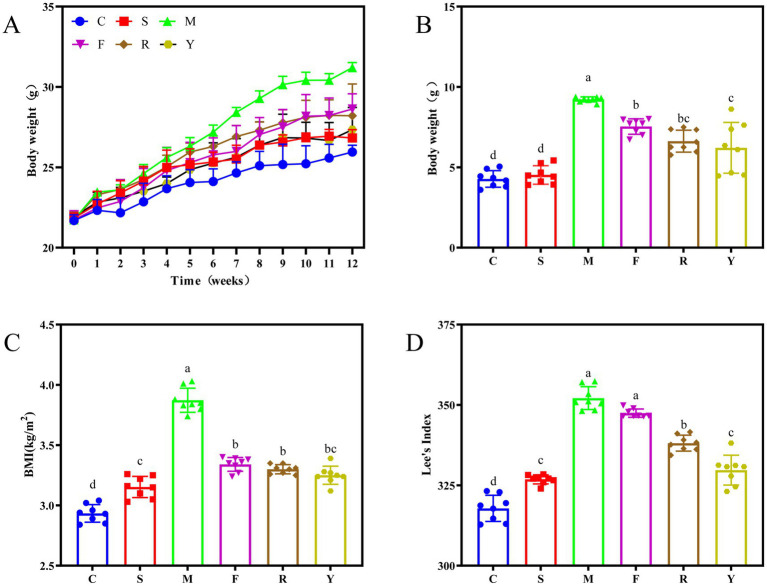
Effect of LAB-fermented broccoli stem by-products on weight-related parameters in mice with diet-induced obesity. **(A)** Overall body weight; **(B)** body weight gain; **(C)** body mass index (BMI); and **(D)** Lee’s index. C: control group, mice fed low-fat diet; M: model group, mice fed HFD; S: positive control group, mice fed HFD and received 60 mg/kg of orlistat; F: fresh BsBP group, mice fed HFD and received 800 mg/kg of fresh BsBP; Y: *Lb. casei* YC5-fermented BsBP group, mice fed HFD and received 800 mg/kg of YC5-fermented BsBP; R: *P. pentosaceus* RBHZ36-fermented BsBP group, mice fed HFD and received 800 mg/kg of RBHZ36-fermented BsBP. Values are expressed as mean ± standard deviation (SD). Groups marked with different superscript letters differ significantly (*p* < 0.05).

To investigate the effects of fermented BsBP on obesity triggered by a HFD, we measured body weight, body mass index (BMI), and Lee’s index in mice receiving the treatment. Body weight progressively increased across all groups with prolonged feeding duration; however, the M group began to show a markedly greater weight gain than the others starting from the sixth week of HFD administration (Figure 2A). By the conclusion of the 12 weeks, the weight gain recorded in the C was 4.27 ± 0.45, while the M group reached 9.30 ± 0.26 g. The significant difference (*p* < 0.05; Figure 2B) in weight gain between these groups confirmed that the obesity model was successfully established. By contrast, mice receiving fermented BsBP showed notably lower weight gain compared to those in the M group, with reductions of 28.7% in the R group and 33.2% in the Y group. Interestingly, BsBP fermented by *Lb. paracasei* YC5 resulted in a more pronounced weight loss effect compared to BsBP fermented by *P. pentosaceus* RBHZ36 (*p* < 0.05). In addition, the BMI and Lee’s index are established metrics for assessing obesity and metabolic health in mice (21). Compared to the M group, both the fresh and fermented BsBP intervention exhibited significant reductions in BMI and Lee’s index (*p* < 0.05; Figures 2C,D). Animals treated with fermented BsBP (R and Y groups) displayed greater improvements in body weight, BMI, and Lee’s index than those receiving unfermented BsBP (F group). Importantly, the outcome in the Y group, which received *Lb. paracasei* YC5-fermented BsBP was comparable to that observed in the positive control group administered orlistat (S group). Collectively, these findings suggest that BsBP fermented with LAB offers substantially greater benefits in curbing the progression of obesity than its fresh counterpart.

### 3.2 Effect of fermented BsBP on liver and adipose coefficients of HFD-fed mice

**Figure 3:**
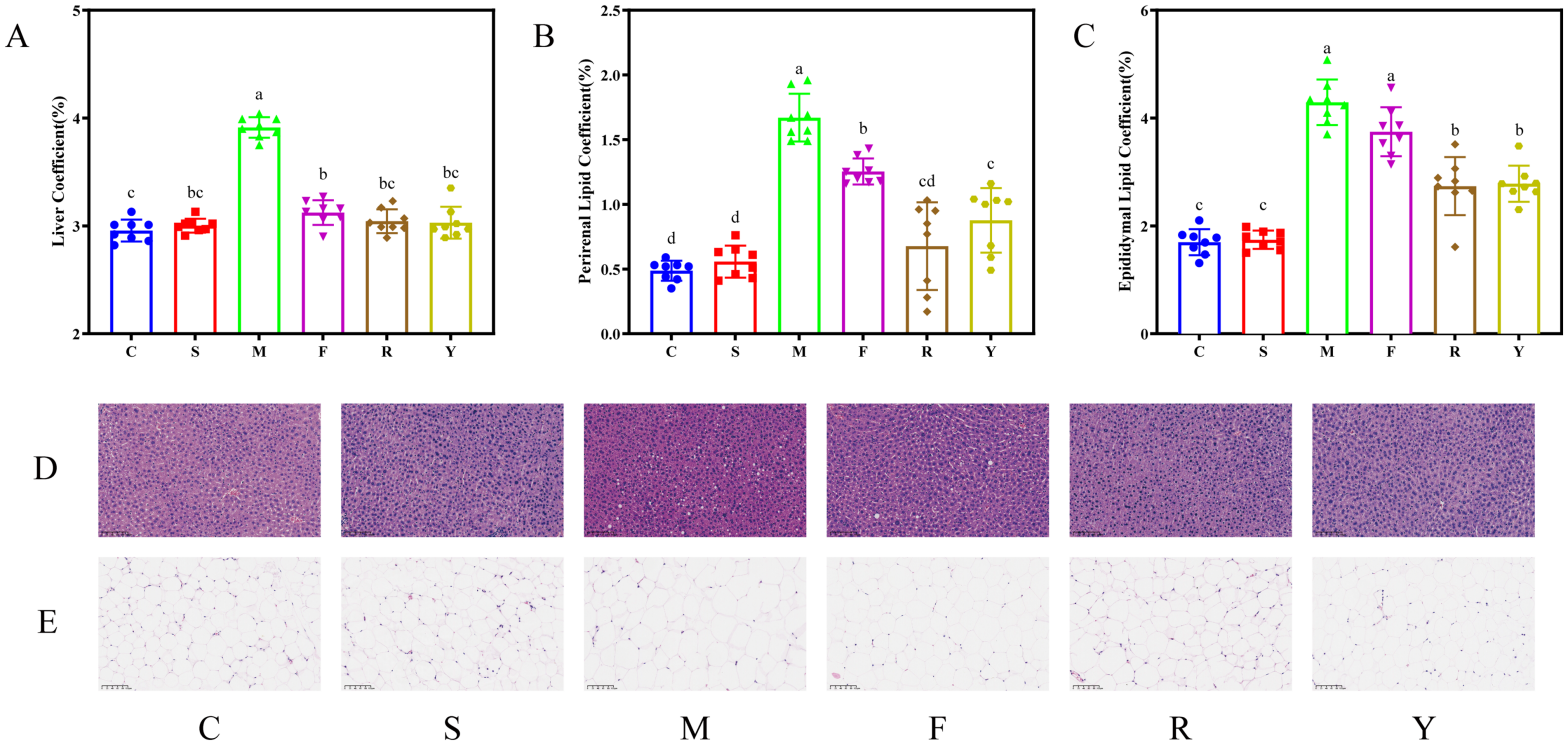
Influence of fermented broccoli stem by-products on liver and adipose tissue indices and histological alterations in high-fat diet (HFD)-induced obese mice. **(A)** Liver-to-body weight ratio; **(B)** perirenal fat index; **(C)** epididymal fat index; **(D)** liver tissue morphology assessed by hematoxylin and eosin (H&E) staining; and **(E)** histological evaluation of epididymal adipose tissue via H&E staining. C: control group, mice fed low-fat diet; M: model group, mice fed HFD; S: positive control group, mice fed HFD and received 60 mg/kg of orlistat; F: fresh BsBP group, mice fed HFD and received 800 mg/kg of fresh BsBP; Y: *Lb. casei* YC5-fermented BsBP group, mice fed HFD and received 800 mg/kg of YC5-fermented BsBP; R: *P. pentosaceus* RBHZ36-fermented BsBP group, mice fed HFD and received 800 mg/kg of RBHZ36-fermented BsBP. Values are expressed as mean ± standard deviation (SD). Groups marked with different superscript letters differ significantly (*p* < 0.05).

The HFDs elevate liver and fat tissue mass ratios in mice (22). After the 12-week intervention, animals in the M group displayed significantly increased liver, perirenal, and epididymal fat coefficients compared to the C group (*p* < 0.05; Figure 3), with the values of 1.32-fold, 3.42-fold, and 2.52-fold higher than those in the C group, respectively. By contrast, administration of either fresh or fermented BsBP significantly attenuated these elevations in tissue mass ratios compared to the M group (*p* < 0.05). These reductions in liver and adipose indices are likely contributing factors to the observed suppression of body weight gain in the intervention groups (23). Groups receiving fermented BsBP (R and Y) demonstrated greater effects on liver and adipose coefficients than the group treated with fresh BsBP (F group), whereas no significant differences were observed in the adipose coefficients between the Y and R groups (*p* > 0.05). This effect is likely associated with the increased production of isothiocyanate-derived compounds, such as sulforaphane (SFN), indole-3-carbinol (I3C), and ascorbigen, generated during the fermentation of BsBP with *Lb. paracasei* YC5 or *P. pentosaceus* RBHZ36 (19). ITC substances, such as SFN and I3C, have the potential to prevent and alleviate HFD-induced symptoms, including epididymal and abdominal fat deposition in mice (11, 13). In fresh broccoli, ITC substances are usually found in small amounts. Instead, their precursors, glucosinolates and the enzyme myrosinase, which is needed for their transformation into active compounds, are compartmentalized in different cellular regions. Fermentation disrupts this separation, leading to a substantial increase in SFN levels, as detected in broccoli puree (24). During fermentation, a substantial increase in SFN content was observed in broccoli puree (25). As a result, the enhanced functional ITC ingredients in fermented BsBP may be attributed to the lowering of liver and adipose coefficients in obese mice.

### 3.3 Effect of fermented BsBP on liver and adipose histology of HFD-fed mice

Histopathological analysis revealed distinct alterations in organ morphology following long-term HFD exposure (26). Herein, our works provided an assessment of the impact of fermented BsBP on organ pathology in obese mice. Microscopic analysis of liver sections showed that hepatocytes in the C group were orderly arranged with intact morphology, displaying a radiating cord-like architecture around central veins, and showed no edema, steatosis, or focal inflammatory infiltration (Figure 3D). By contrast, M group exhibited disorganized hepatic lobules, marked macrovesicular steatosis characterized by large lipid droplets displacing hepatocyte nuclei to the cytoplasmic periphery, and pronounced inflammatory infiltration. However, both positive control treatment (S group) and broccoli stem intervention (F, R, and Y groups) significantly ameliorated hepatic tissue damage, as evidenced by restored lobular architecture and reduced pathological features. In addition, histological evaluation of epididymal adipose tissue via H&E staining demonstrated that adipocytes in the C group were densely packed and uniformly small, with cell diameters significantly smaller than those in the M group (Figure 3E). Compared to the M group, both fresh (F group) and fermented BsBP (R and Y group) exhibited significant amelioration in adipocyte hypertrophy. Notably, fermented BsBP with *Lb. paracasei* YC5 treatment (Y group) achieved the most pronounced reduction in adipocyte size, closely resembling that of the C group. Our results align with previous studies that long-term HFD has detrimental effects on hepatic and epididymal adipose tissues (23). However, these changes were effectively ameliorated by fermented BsBP treatment, demonstrating comparable efficacy to positive control treatments for liver and adipose histology.

### 3.4 Effect of fermented BsBP on serum biochemical indicators of HFD-fed mice

**Figure 4:**
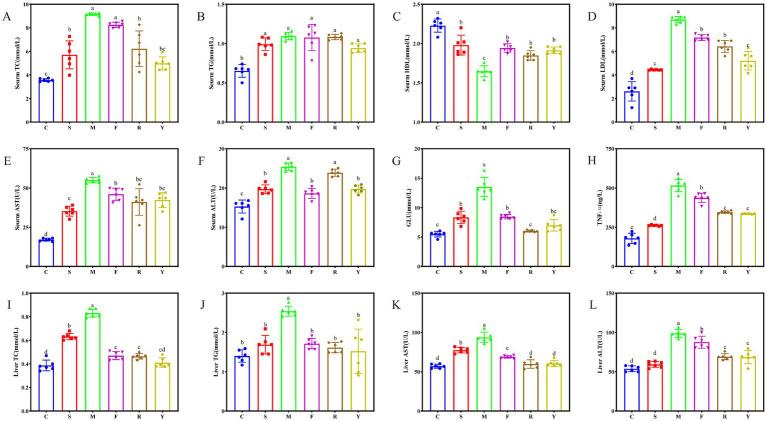
Effect of fermented BsBP on serum lipids, blood glucose, and liver function of HFD-induced obese mice. **(A)** Total cholesterol (TC) in serum; **(B)** serum triglycerides (TG); **(C)** serum aspartate aminotransferase (AST); **(D)** serum alanine aminotransferase (ALT); **(E)** high-density lipoprotein cholesterol (HDL-C); **(F)** low-density lipoprotein cholesterol (LDL-C); **(G)** blood glucose concentration; **(H)** hepatic tumor necrosis factor-alpha (TNF-*α*); **(I)** total cholesterol in liver; **(J)** hepatic triglycerides; **(K)** liver AST activity; and **(L)** liver ALT activity. C: control group, mice fed low-fat diet; M: model group, mice fed HFD; S: positive control group, mice fed HFD and received 60 mg/kg of orlistat; F: fresh BsBP group, mice fed HFD and received 800 mg/kg of fresh BsBP; Y: *Lb. casei* YC5-fermented BsBP group, mice fed HFD and received 800 mg/kg of YC5-fermented BsBP; R: *P. pentosaceus* RBHZ36-fermented BsBP group, mice fed HFD and received 800 mg/kg of RBHZ36-fermented BsBP. Values are expressed as mean ± standard deviation (SD). Groups marked with different superscript letters differ significantly (*p* < 0.05).

Long-term HFD also induced dysregulation of serum lipids in mice, resulting in a remarkable increase in serum TC, TG, and LDL-C, alongside serum liver enzyme activities (AST, ALT) (27) (Figures 4A–F). At the end of the 12-week intervention period, mice in the M group exhibited significantly elevated levels of serum TC, TG, and liver enzymes AST and ALT levels compared to the C group mice (*p* < 0.05). Fermented BsBP intervention groups (R and Y) showed a marked reduction in serum TC relative to the M group, with reductions of 31.9% in the R group and 45.5% in the Y group. Moreover, TC concentrations in the Y group were similar to those of the C group. By contrast, mice receiving the unfermented BsBP (F group) did not exhibit a significant change in TC levels compared to the M group (*p* > 0.05), indicating that the fresh form had a minimal impact on cholesterol regulation. Furthermore, TG levels in serum did not differ significantly among the S, F, R, and Y groups when compared to the M group (*p* > 0.05; Figure 4B).

Long-term HFD leads to low serum HDL-C levels and high LDL-C levels (28). Consistent with previous studies, mice in the M group fed an HFD displayed a marked decrease in HDL-C and a significant increase in LDL-C levels (*p* < 0.05; Figures 4C,D). The positive control treatment and fermented BsBP interventions showed significant effects. Notably, the Y group exhibited a particularly remarkable impact, with a 14.7% increase in HDL-C and a 40.1% reduction in LDL-C levels in the serum. Interestingly, fresh and fermented BsBP showed similar effects on inhibiting serum AST and ALT elevation in HFD-fed mice (see Figures 4E,F), which was unlike the alleviating effect on TC and LDL-C levels. Specifically, the Y group experienced a 23.1% decrease, and the F group experienced a 16.5% decrease in AST levels compared to the M group. In terms of ALT levels, the Y group decreased by 22.3%, while the F group experienced a 28.3% decrease. The reduction in AST within the Y group was greater than in the F group, whereas the reduction in ALT was not as significant as in the F group. Blood glucose levels showed that HFD caused abnormal glucose metabolism, while fresh and fermented BsBP significantly mitigated this impairment (*p* < 0.05) (Figure 4G); moreover, BsBP fermented with LAB yielded a more favorable outcome, with reductions of 55.5% in the R group and 48.2% in the Y group. Collectively, a long-term HFD significantly altered serum lipid and blood glucose levels, while fermented BsBP with LAB intervention effectively attenuated these abnormalities.

### 3.5 Effect of fermented BsBP on liver biochemical indicators of HFD-fed mice

The liver plays a crucial role in lipid synthesis, encompassing lipid uptake, storage, and distribution. However, long-term HFD exposure induces hepatic injury by disrupting these metabolic processes (29). In this study, we analyzed liver biochemical indicators, including TNF-*α*, TC, TG, AST, and ALT, to investigate the alleviating effect of fermented BsBP on liver injury in HFD-fed mice. The 12-week HFD diet administration increased hepatic TNF-α levels in the M group (Figure 4H; *p* < 0.05). However, such trends were remarkably relieved by the intervention of fermented BsBP, with reductions of 33.3% in the R group and 35% in the Y group. TC, TG, AST, and ALT levels in the liver were also significantly elevated in the M group compared to the C group (*p* < 0.05), indicative of liver injury (Figures 4I–L) (30). However, positive control treatment and BsBP interventions reduced hepatic TC, TG, AST, and ALT levels as opposed to the M group. Similar to the above results, fermented BsBP with LAB (Y and R groups) exhibited a more substantial reduction in these indicators compared to F group, approaching the levels of the C group. Fermented BsBP interventions resulted in a reduction in hepatic TC, TG, AST, and ALT levels ranging from 44.3–50.6%, 36.5–40.2%, 35.3–36.2%, and 30.1–30.6%, respectively. These results suggest that fermented BsBP exhibits greater improvement than fresh BsBP in ameliorating HFD-induced TC and TG accumulation, hepatic injury, and inflammatory responses in mice. These increased effects are potentially linked to the action of the probiotic strains used during fermentation.

### 3.6 Effect of fermented BsBP on gut microbiota of HFD-fed mice

The gut microbiota contributes to the onset and progression of metabolic diseases, including diabetes, obesity, and hepatic steatosis (31). Emerging studies have emphasized that the consumption of probiotic-fermented foods can effectively mitigate obesity by restoring the dysbiotic gut microbiota (22, 23). In the current study, fermented BsBP with LAB was administered to obese mice as an intervention, and diversity and gut microbiota composition analysis were performed on gut microbiota sequencing data to assess microbial community structure.

**Figure 5:**
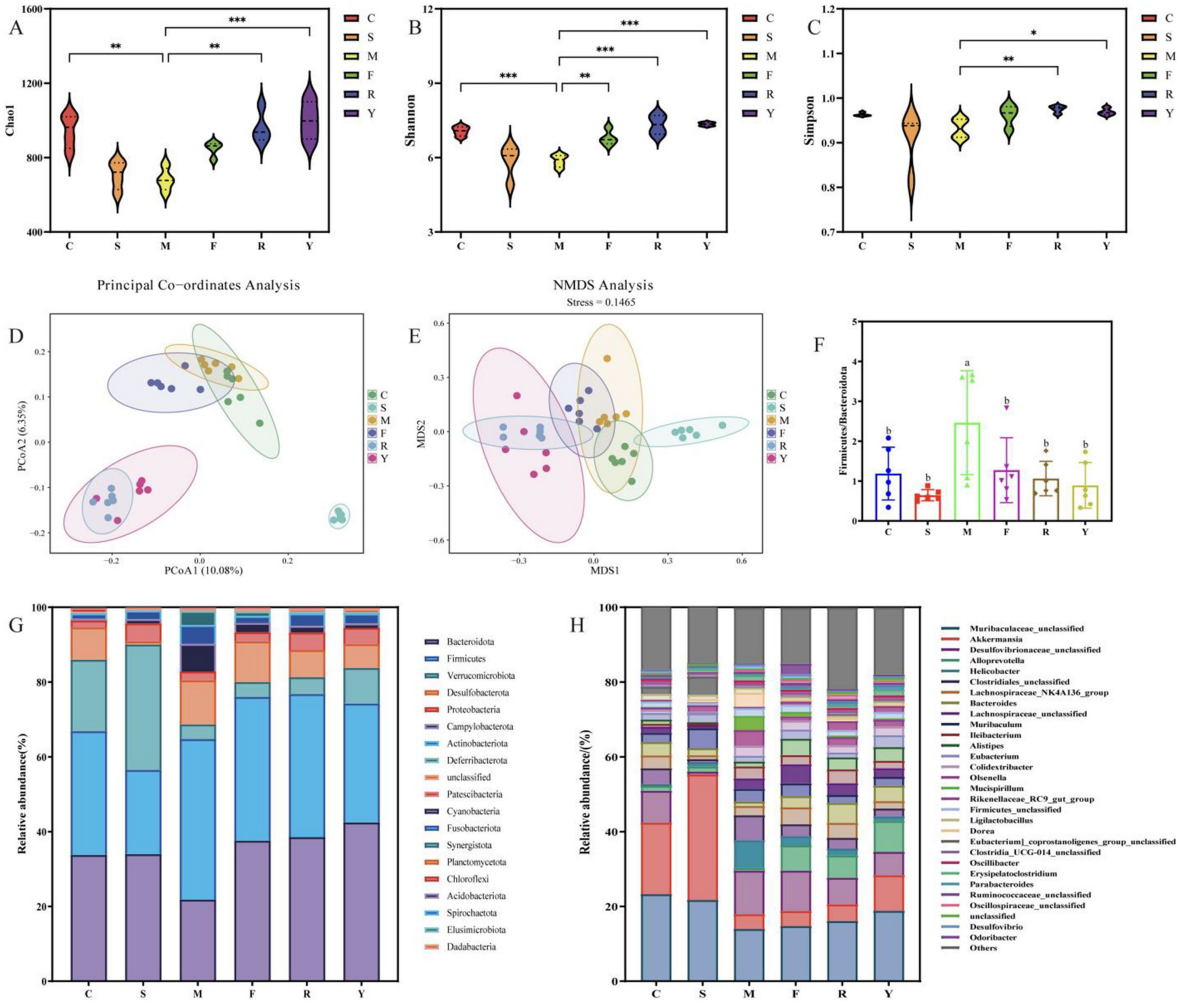
Modulatory effects of BsBP on gut microbiota in mice with HFD-induced obesity. **(A)** Chao1 richness estimator; **(B)** Shannon diversity index; **(C)** Simpson index; **(D)** principal coordinates analysis (PCoA); **(E)** non-metric multidimensional scaling (NMDS); **(F)** Firmicutes to Bacteroidota (F/B) ratio; **(G)** relative abundance of bacterial phyla; and **(H)** genus-level microbial composition. C: control group, mice fed low-fat diet; M: model group, mice fed HFD; S: positive control group, mice fed HFD and received 60 mg/kg of orlistat; F: fresh BsBP group, mice fed HFD and received 800 mg/kg of fresh BsBP; Y: *Lb. casei* YC5-fermented BsBP group, mice fed HFD and received 800 mg/kg of YC5-fermented BsBP; R: *P. pentosaceus* RBHZ36-fermented BsBP group, mice fed HFD and received 800 mg/kg of RBHZ36-fermented BsBP. Values are expressed as mean ± standard deviation (SD). Groups marked with different superscript letters differ significantly (*p* < 0.05).

A total of 2,369,323 effective tags were generated from 36 fecal sample (Supplementary Table S3), and sequencing depth was deemed to be sufficient (Supplementary Figure S1). Analysis of alpha diversity revealed a significant decline in both Chao1 and Shannon indices in the M group compared to the C group (*p* < 0.05), indicating a substantial reduction in microbial richness among mice fed an HFD (Figures 5A–C). This observation aligns with earlier research indicating that high-fat dietary intake compromises gut microbial stability, resulting in diminished diversity and overall richness (23). The fresh BsBP intervention (F group) could modestly increase gut microbiota species richness, with a slight elevation in the Shannon index. Notably, the fermented BsBP intervention (R and Y groups) induced significant improvements in microbial diversity (*p* < 0.05 for the Chao1 and Shannon indices), with the Y group exhibiting the most pronounced increase in diversity. Moreover, differences in gut microbial community structure were evaluated using principal coordinate analysis (PCoA) along with non-metric multidimensional scaling (NMDS) methods. The M and C groups exhibited distinct clustering, with only minimal overlap in their confidence ellipses, indicating that the HFD resulted in substantial alterations to the gut microbial structure (Figures 5D,E). Following intervention with the positive control and BsBP treatment, all treatment groups showed varying degrees of divergence from the M group, with the Y group demonstrating the most pronounced deviation.

At the phylum level, deeper taxonomic profiling showed that the predominant microbial groups across all experimental mice included Bacteroidota, Firmicutes, Verrucomicrobiota, Desulfobacterota, and Proteobacteria (Figure 5G). Compared to the M Group, all intervention groups exhibited increased relative abundances of Bacteroidota and Verrucomicrobiota, while the relative abundance of Firmicutes was reduced. The Firmicutes/Bacteroidota ratio (F/B), a critical biomarker for health assessment, is inversely correlated with health status, with higher values indicating poorer metabolic health (32, 33). Chen et al. (34) demonstrated the positive role of Firmicutes in adipose accumulation, while studies have proposed that Bacteroidota actively promotes lipid catabolism. The Firmicutes-to-Bacteroidota ratio was significantly higher in the M group than in the C group (*p* < 0.05; Figure 5F). After treatment with either the positive control or BsBP formulations, the Firmicutes/Bacteroidota ratio declined substantially, and approached levels comparable to those observed in the C group, with no statistically significant difference (*p* > 0.05).

**Figure 6:**
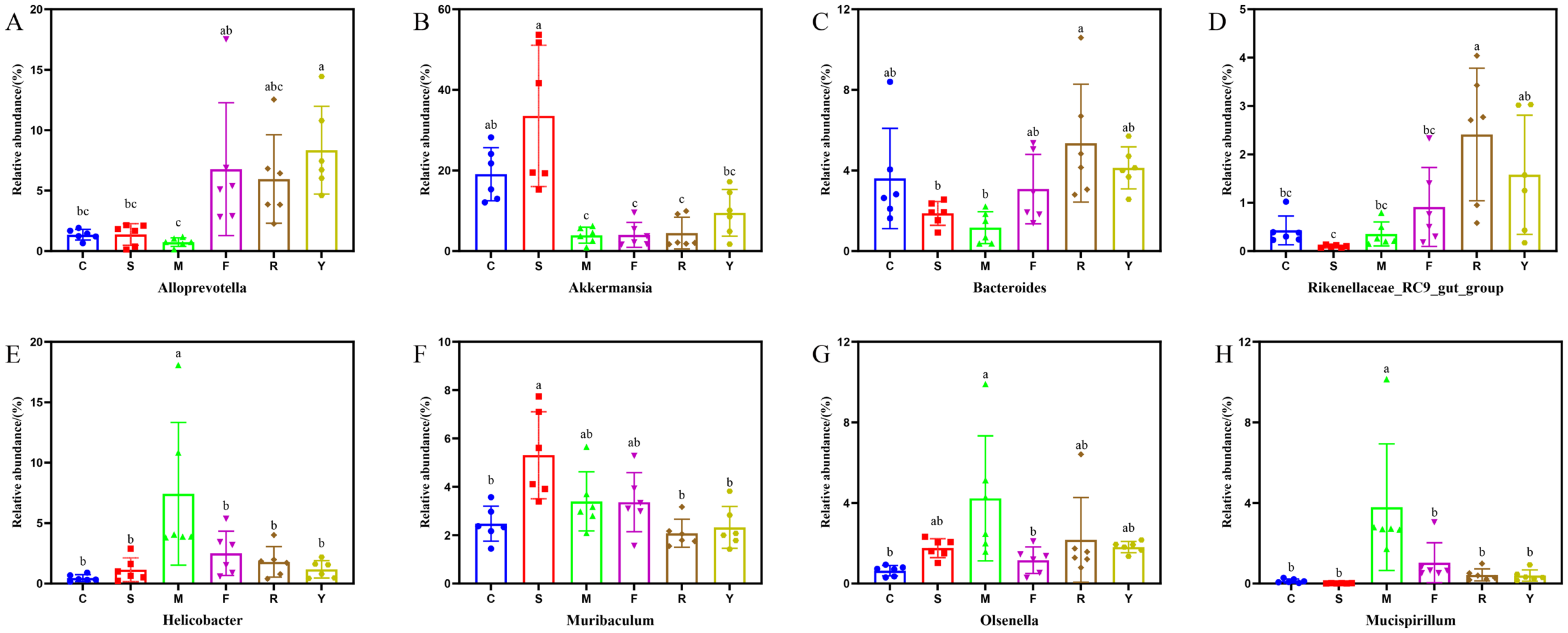
Effect of fermented BsBP on the relative abundance of individual genus. **(A)** *Alloprevotella*; **(B)** *Akkermansia*; **(C)** *Bacteroides*; **(D)** *Rikenellaceae_RC9_gut_group*; **(E)** *Helicobacter*; **(F)** *Muribaculum*; **(G)** *Olsenella*; and **(H)** *Mucispirillum*. C: control group, mice fed low-fat diet; M: model group, mice fed HFD; S: positive control group, mice fed HFD and received 60 mg/kg of orlistat; F: fresh BsBP group, mice fed HFD and received 800 mg/kg of fresh BsBP; Y: *Lb. casei* YC5-fermented BsBP group, mice fed HFD and received 800 mg/kg of YC5-fermented BsBP; R: *P. pentosaceus* RBHZ36-fermented BsBP group, mice fed HFD and received 800 mg/kg of RBHZ36-fermented BsBP. Values are expressed as mean ± standard deviation (SD). Groups marked with different superscript letters differ significantly (*p* < 0.05).

Genus-level profiling revealed that the dominant bacterial taxa among all groups included unclassified members of *Muribaculaceae, Desulfovibrionaceae*, *Akkermansia*, *Alloprevotella*, *Lachnospiraceae_NK4A136_group*, and *Bacteroides* (Figure 5H). Consistent with recent research, our data indicate that prolonged consumption of an HFD produces significant disturbances to gut microbial equilibrium (22, 23). Compared to the C group, the M group exhibited significantly increased relative abundances of harmful bacterial genera, including *Desulfovibrionaceae unclassified*, *Helicobacter*, *Clostridiales unclassified,* and *Muribaculum*. By contrast, the relative abundances of beneficial genera (e.g., *Muribaculaceae unclassified*, *Akkermansia,* and *Alloprevotella*) were substantially reduced. Following interventions with the BsBP, *Akkermansia, Bacteroides*, *Alloprevotella*, *Rikenellaceae_RC9_gut_group*, and *Lachnospiraceae unclassified*, the relative abundances increased compared to the M group; however, *Helicobacter*, *Muribaculum*, *Olsenella*, and *Mucispirillum* showed significant reductions in their relative abundances. Notably, *Akkermansia* and *Alloprevotella* were significantly enriched by *Lb. paracasei* YC5 fermented BsBP, increasing from 3.9 to 9.5% and 0.7 to 8.3%, respectively; whereas *Bacteroides* were significantly enriched by *P. pentosaceus* RBHZ36 fermented BsBP, rising from 1.2 to 5.4%. Meanwhile, the fermented BsBP intervention groups (R and Y) exhibited a 5.6–6.3% decrease in *Helicobacter* compared to the M group, while these changes were less pronounced for fresh BsBP. The microbial functions predicted by PICRUSt showed that fermented BsBP altered the functional profiles of the gut microbiota (Figure 6). Fermented BsBP with *Lb. paracasei* YC5 (Y group) intervention significantly increased the relative abundance of microbial genes associated with energy metabolism and PPAR signaling pathway, which may contribute to the effective prevention of obesity. Herein, PICRUSt only provides putative functions; therefore, further research is needed to clarify their relationship.

### 3.7 Correlation between gut microbiota and obesity-related biochemical indices

**Figure 7:**
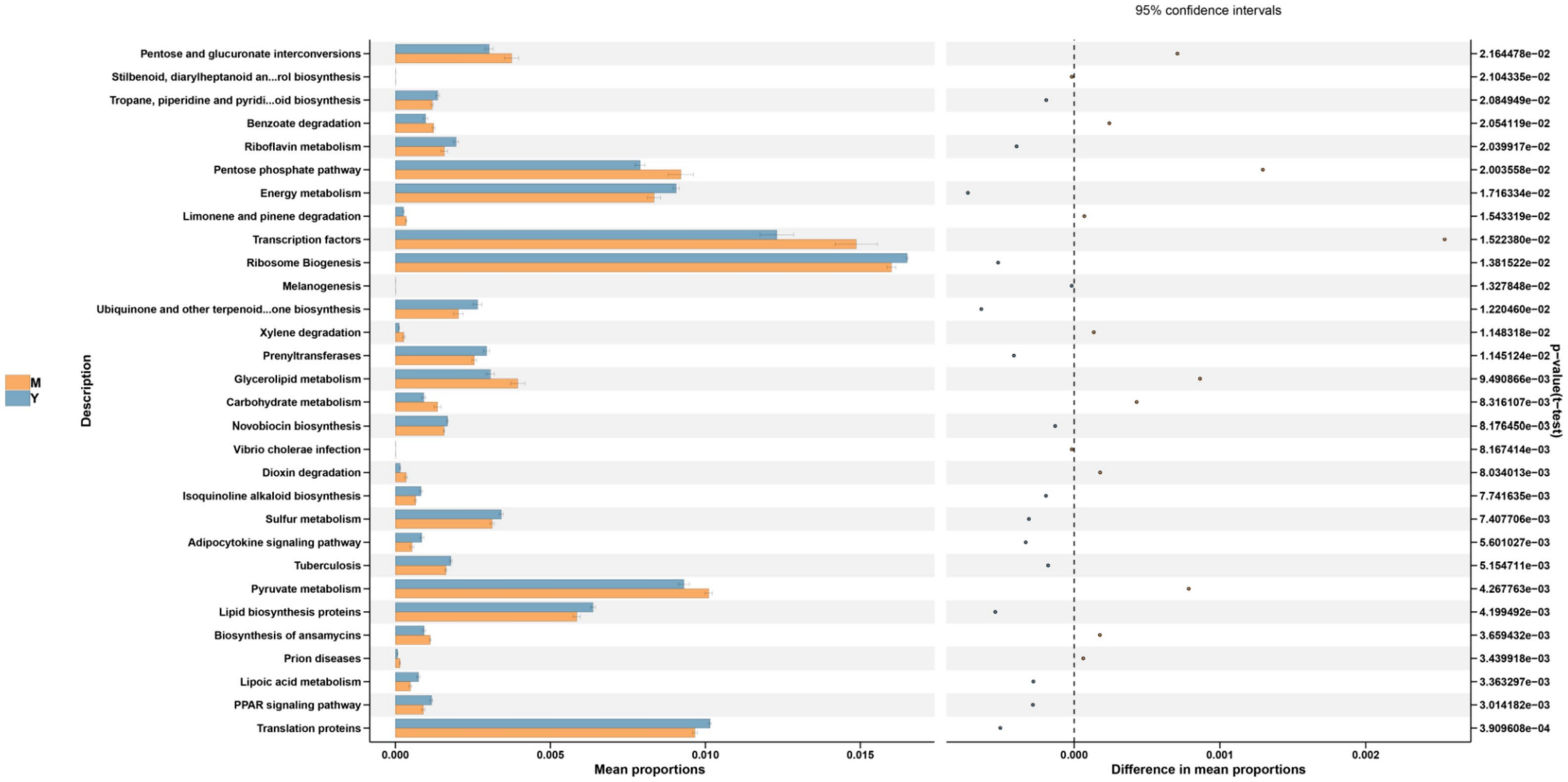
Functional prediction (PICRUSt) of the gut microbiota based on KEGG pathways in M and Y group. M group: model group, mice fed HFD; Y group: *Lb. casei* YC5-fermented BsBP group, mice fed HFD and received 800 mg/kg of YC5-fermented BsBP.

**Figure 8:**
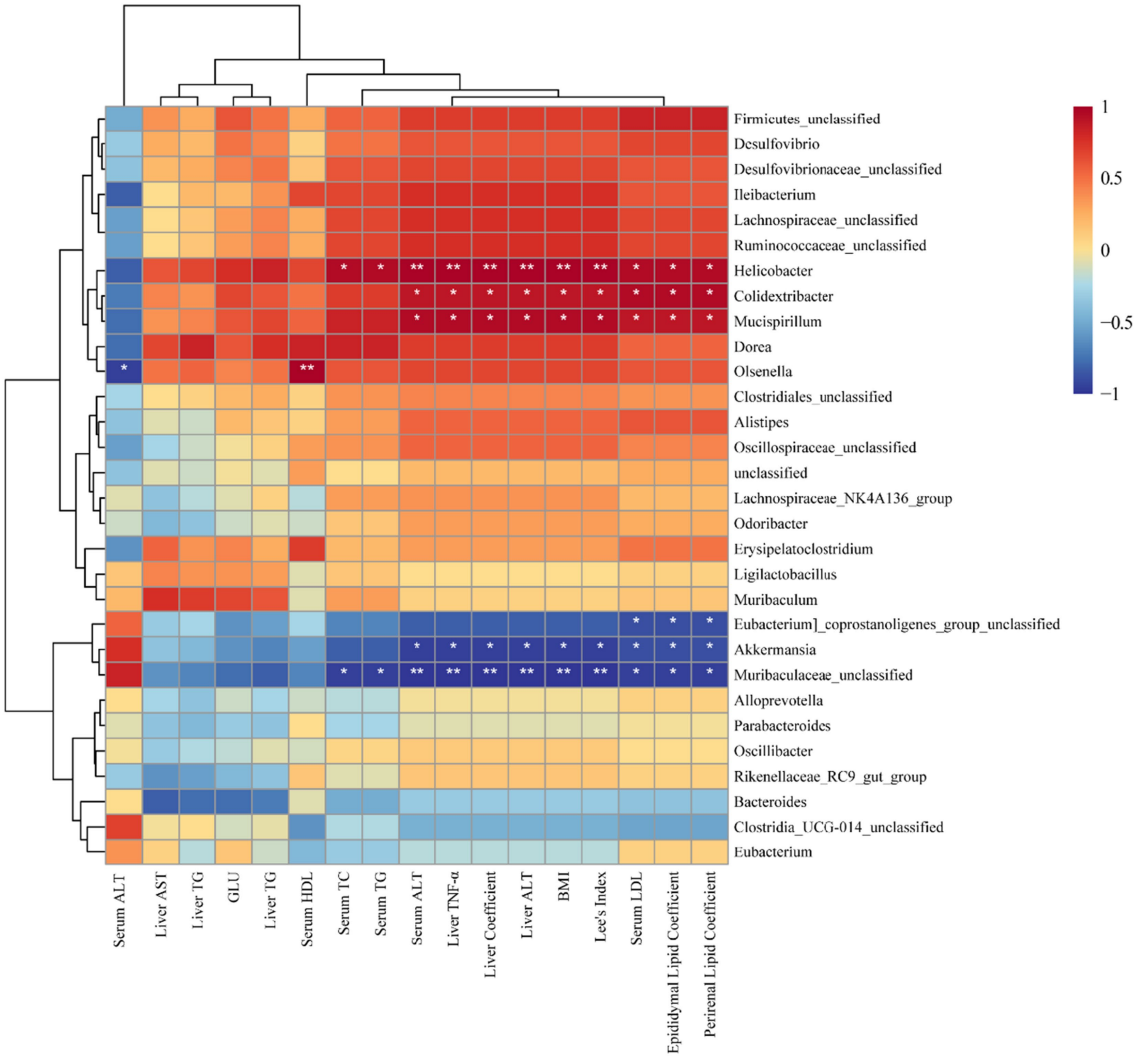
Spearman correlation heatmap illustrating the associations between key gut microbial genera and obesity-related biochemical indices. Statistically significant correlations are indicated as follows: *p < 0.05, **p < 0.01, and ***p < 0.001.

Finally, the relationship between dominant gut bacterial genera and obesity-related biochemical indices was estimated using Spearman’s rank correlation. A heatmap was generated to visualize the results of correlation clustering analysis (Figure 7). Within the dominant gut microbiota genera, *Helicobacter*, *Colidextribacter*, *Mucispirillum,* and *Olsenella* exhibited predominantly positive correlations with obesity-related factors. High levels of *Colidextribacter* strongly correlate with clinical indicators of obesity (35), which aligns with the findings of our study. In addition, *Helicobacter* has a bidirectional relationship with obesity (36), and our analysis revealed a significant and positive correlation between its abundance and increased body weight. Furthermore, *Mucispirillum* is recognized for its ability to induce intestinal inflammation and has been associated with chronic conditions such as Crohn’s disease and ulcerative colitis (37); our work demonstrated significant positive correlations with multiple obesity-related factors. In the present study, fermented BsBP suppressed these obesity-related genera and facilitated the growth of *Muribaculaceae unclassified, Akkermansia, Alloprevotella,* and *Bacteroides.* By contrast, these genera exhibited negative correlations with obesity-related biochemical indices (Figure 7). It is worth emphasizing that the genus *Akkermansia*, a beneficial microbial taxon, exhibits anti-obesity effects and is significantly negatively correlated with body weight. Recent studies have indicated that *Akkermansia* can reinforce the intestinal barrier, produce short-chain fatty acids, and regulate immune responses and appetite hormones, ultimately exhibiting a significant inverse correlation with body weight (38, 39). Additionally, *Akkermansia* upregulates genes involved in PPAR signaling pathways, such as CPT1, PGC-1α, and PPARα (40), a finding that aligns with our microbial functional prediction data (Figure 6). Besides, the increase in *Bacteroides* abundance within the fermented BsBP group may also contribute to alleviating obesity. Previous studies have shown that supplementation with various *Bacteroides* species, including *B. vulgatus*, *B. uniformis*, and *B. acidifaciens*, have the effect of reducing weight and alleviating metabolic dysfunction (41, 42). The anti-obesity effects of *Bacteroides* are linked to the promotion of fatty tissue oxidation via the bile acid axis and the regulation of branched-chain amino acid metabolism (43, 44). As a result, administering fermented BsBP helped restore the gut microbial balance disrupted by HFD intake, promoting the proliferation of health-associated genera, such as *Akkermansia* and *Bacteroides*, while reducing the abundance of taxa linked to obesity, including *Colidextribacter*, *Helicobacter*, and *Mucispirillum*. These findings were consistent with previous studies, which showed that broccoli cloud regulates the gut microbiota, increasing *Bacteroides and Akkermansia* and reducing *Mucispirillum* abundance, in which ITCs contributed to this effect (40, 45) (Figure 8).

The BsBPs are usually discarded as food waste. In the present study, BsBPs were fermented using two LAB strains (*Lb. Casei* YC5 and *P. pentosaceus* RBHZ36), resulting in an elevated generation of bioactive ITCs metabolites, which may contribute to reducing lipid accumulation in obese mice. This study aims to valorize these by-products into valuable functional foods, thereby reducing waste and enhancing sustainability in the food industry. In addition, this research indicates significant improvements in weight gain, adipose tissue mass, serum lipid profiles, glucose levels, liver function markers, and inflammatory indicators in mice treated with LAB-fermented BsBP compared to those given fresh BsBP. Our findings provide an evidence that LAB-fermented BsBP might be a promising functional food for obesity management. However, there are some limitations of this study. Although ITCs (sulforaphane, I3C) were enhanced in LAB-fermented BsBPs, the individual contributions of these ITCs to the regulation of lipid metabolism and gut microbial composition were not explored. The direct quantification of ITCs in the serum or tissues of mice should be further conducted. In addition, we employed predictive functional analysis (PICRUSt) instead of gene expression validation to infer the microbial functions, thus limiting the precision of these inferences. Further gene expression are needed to confirm our results in the future.

## 4 Conclusion

The BsBP fermented with *Lb. casei* YC5 or *P. pentosaceus* RBHZ36 (corresponding to groups Y and R) demonstrated greater anti-obesity efficacy than the unfermented form (F group), as evidenced by reductions in body weight gain, fat tissue indices, and improvements in serum lipid profiles and blood glucose levels. These treatments also alleviated liver damage induced by HFD consumption. Moreover, the intervention altered the gut microbial community, promoting the proliferation of health-associated genera, such as *Akkermansia* and *Bacteroides*. Collectively, these findings support the potential of LAB-fermented BsBP as a promising functional food for obesity management. Nonetheless, further research is needed to elucidate the precise roles of the resulting bioactive metabolites in mediating these beneficial effects.

## Data Availability

The original data presented in the study are included in the article/supplementary material, and the data presented in the study are deposited in the SRA repository, accession number PRJNA1332987 (https://www.ncbi.nlm.nih.gov/bioproject/PRJNA1332987).

## Footnotes

^1^ https://www.omicstudio.cn/tool
