# Supplementary material for: Fermented broccoli stalk by-product with lactic acid bacteria ameliorates high-fat diet-induced obesity in C57BL/6 mice
Source: Front Nutr. 2025 Oct 9;12:1670009. doi: 10.3389/fnut.2025.1670009 (PMC12544999; doi:10.3389/fnut.2025.1670009)
Supplement: Supplementary file 1 [file Table_1.DOCX]

**Supporting Information**

**Fermented broccoli stalk by-product with lactic acid bacteria ameliorates high-fat diet-induced obesity in C57BL/6 mice**

Lihao Jiang^a,b^, Qinghang Wu^b^, Zhiyi Lu^c^, Jianming Zhang^b^, Dandan Zhao^c^, Daqun Liu^b,^*, Chengcheng Zhang^b,^*

^a^ College of Biological and Environmental Sciences, Zhejiang Wanli University, Ningbo, 315100, China

^b^ Food Science Institute, Zhejiang Academy of Agricultural Sciences, Hangzhou 310021, China

^c^ Ecology and Health Institute, Hangzhou Vocational & Technical Collge, Hangzhou, 310018, China

*Corresponding author:

Daqun Liu, *E-mail address*: liudaqun@zaas.ac.cn

Chengcheng Zhang, *E-mail address*: zhangcc@zaas.ac.cn

***Nutritional ingredient analysis:*** The moisture, ash, protein, fat, and carbohydrates in fermented broccoli stalk by-product (BsBP) were determined according to Chinese National Standards GB 5009.3–2016, GB 5009.4–2016, GB 5009.5–2016, GB 5009.6–2016, and GB/T 15672–2009, respectively.

***GSLs analysis:*** GSLs in fermented BsBP were analysed according to our previous method (Qinghang et al., 2023). In short, the lyophilized powder sample (0.5 g) was mixed with 5 mL 70% methanol for 15 min at 70°C. Then, ultrasonic extraction was performed at room temperature for 30 min follow by centrifugation at 13,000 rpm for 5 min. The supernatants were pooled and diluted to 15 mL, and filtered with a 0.22 µm filter. The quantitative analysis of GSL content was carried out via ultra-high performance liquid chromatography-triple quadrupole mass spectrometry (UPLC-QqQ-MS/MS). Chromatographic separation was performed on a ZORBAX C18 column (3.0 × 100 mm, 1.8 µm). Multiple reaction monitoring (MRM) of selected ions in the first (Q1) and third quadrupole (Q3) was carried out in negative ion mode. GSLs were quantified using the calibration curves of aliphatic glucoraphanin.

***GSL degradation products analysis:*** The GSL degradation products (SFN, I3C, and ascorbigen) in fermented BsBP were analysed according to our previous method (Qinghang et al., 2023). 50 mg of a freeze-dried powder were extracted by 5 mL of distilled water for 2 h. After centrifugation (15,000 rpm for 5 min), the supernatant was collected, diluted to 5 mL, and then filtered with a 0.22 µm filter for later use. A UPLC-QqQ-MS/MS system, coupled with a ZORBAX C18 column was used to determine the degradation products in the extract. MRM was used to selectively fragment ions from the first quadrupole (Q1) to generate specific daughter ions in the third quadrupole (Q3) in positive ion mode. The GLS degradation products were quantified using their respective standard curves.

**Table S1.** The ingredients of the normal chow diet and high fat diet (HFD).

| LFD（g）  (10% calorie from fat) | | HFD（g）  (45% calorie from fat) | |
| --- | --- | --- | --- |
| Casein | 200.00 | Casein | 200.00 |
| L-Cystine | 3.00 | L-Cystine | 3.00 |
| Sucrose | 68.80 | Sucrose | 172.80 |
| Maltodextrin | 125.00 | Maltodextrin | 100.00 |
| Corn starch | 506.20 | Corn starch | 72.80 |
| Lard | 20.00 | Lard | 177.50 |
| Soybean oil (TBHQ) | 25.00 | Soybean oil (TBHQ) | 25.00 |
| Fibre | 50.00 | Fibre | 50.00 |
| Mineral mix | 10.00 | Mineral mix | 10.00 |
| Calcium carbonate | 5.50 | Calcium carbonate | 5.50 |
| Calcium hydrogen phosphate | 13.00 | Calcium hydrogen phosphate | 13.00 |
| Potassium citrate | 16.50 | Potassium citrate | 16.50 |
| Vitamin Mix | 10.00 | Vitamin Mix | 10.00 |
| Choline tartrate | 2.00 | Choline tartrate | 2.00 |
| Blue food color | 0.01 | Red food color | 0.05 |
| Yellow food color | 0.04 |  |  |

**Table S2** The information for commercial kits.

| **Determination** | **Catalog numbers** | **Assay sensitivity** | **R^2^** |
| --- | --- | --- | --- |
| TC | A111-1-1 | 0～19.39 mmol/L | 0.995 |
| TG | A110-1-1 | 0.3～11.4 mmol/L | 0.995 |
| HDL-C | A112-1-1 | not provide | not provide |
| LDL-C | A113-1-1 | not provide | not provide |
| AST | C010-2-1 | 0～96.4 U/L | 0.999 |
| ALT | C009-2-1 | 0～96.4 U/L | 0.999 |
| TNF-α | H052-1-2 | 3.75～250 ng/L | 0.990 |

**Table. S3** Quality analysis of 16S rRNA gene sequencing data.

| Sample | Valid_Tags | Valid_Bases | Q20% | Q30% | GC% |
| --- | --- | --- | --- | --- | --- |
| C1 | 64980 | 26.97M | 97.46 | 92.92 | 53.87 |
| C2 | 69218 | 28.52M | 97.95 | 94.36 | 54.6 |
| C3 | 70451 | 29.18M | 97.69 | 93.65 | 54.52 |
| C4 | 66023 | 27.18M | 97.39 | 92.9 | 54.2 |
| C5 | 66857 | 27.75M | 97.78 | 93.73 | 53.98 |
| C6 | 71783 | 29.80M | 97.29 | 92.28 | 55.54 |
| S1 | 65684 | 27.25M | 97.76 | 93.72 | 55.12 |
| S2 | 62552 | 25.92M | 97.79 | 93.9 | 54.69 |
| S3 | 71070 | 29.40M | 97.65 | 93.26 | 54.69 |
| S4 | 67486 | 27.90M | 97.41 | 92.63 | 55 |
| S5 | 68508 | 28.25M | 97.57 | 93.15 | 54.47 |
| S6 | 71011 | 29.29M | 96.91 | 91.35 | 54.82 |
| M1 | 65582 | 27.09M | 98.13 | 94.8 | 55.04 |
| M2 | 71718 | 29.87M | 97.67 | 93.37 | 55.17 |
| M3 | 67635 | 28.23M | 98.04 | 94.6 | 54.39 |
| M4 | 65372 | 27.01M | 97.55 | 93.13 | 55.33 |
| M5 | 65515 | 27.05M | 98.15 | 94.79 | 52.69 |
| M6 | 73776 | 30.51M | 98.09 | 94.68 | 55.11 |
| F1 | 64563 | 26.83M | 98.09 | 94.57 | 53.77 |
| F2 | 67214 | 27.83M | 97.4 | 92.67 | 53.91 |
| F3 | 64139 | 26.65M | 97.85 | 94.12 | 53.78 |
| F4 | 74712 | 30.99M | 98 | 94.49 | 53.02 |
| F5 | 64847 | 27.09M | 97.89 | 94.29 | 53.46 |
| F6 | 66212 | 27.35M | 97.79 | 94.1 | 54.07 |
| R1 | 68454 | 28.38M | 96.54 | 90.82 | 54.62 |
| R2 | 61591 | 25.53M | 97.61 | 93.66 | 53.59 |
| R3 | 67499 | 28.21M | 97.64 | 93.66 | 53.02 |
| R4 | 63631 | 26.48M | 97.6 | 93.6 | 53.68 |
| R5 | 59686 | 24.71M | 97.78 | 94.04 | 53.74 |
| R6 | 64532 | 26.77M | 96.46 | 90.68 | 53.99 |
| Y1 | 57799 | 23.87M | 97.46 | 93.2 | 53.26 |
| Y2 | 60359 | 25.00M | 97.49 | 93.43 | 53.82 |
| Y3 | 63738 | 26.38M | 98.02 | 94.52 | 53.72 |
| Y4 | 53308 | 22.08M | 96.59 | 90.97 | 53.81 |
| Y5 | 66090 | 27.52M | 97.56 | 93.48 | 53.63 |
| Y6 | 55728 | 23.08M | 97.3 | 92.89 | 53.89 |


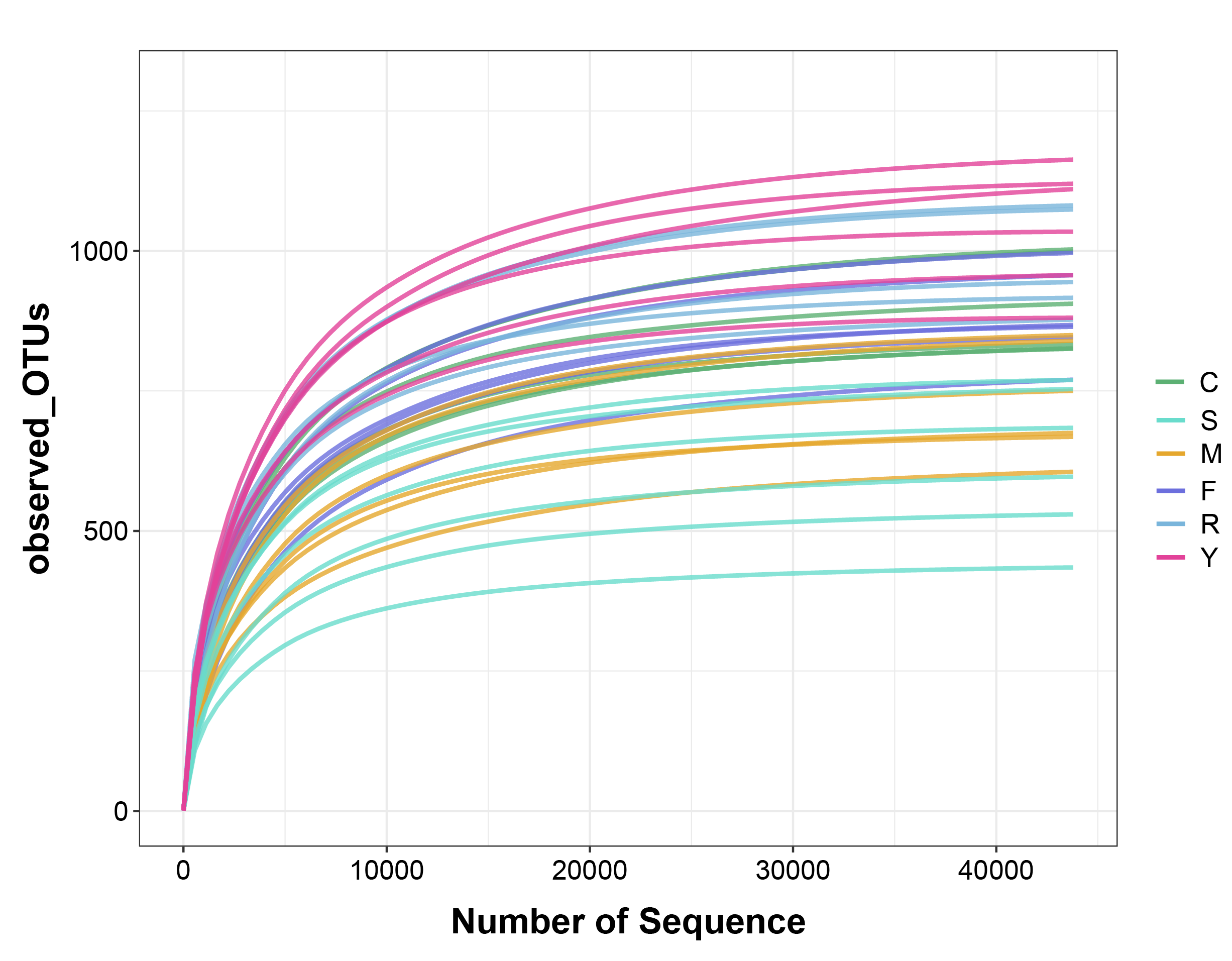


**Figure S1** Rarefaction curve of OTUs.

**References:**

Qinghang, W., Zhang, C., Zhang, J., Xin, X., Li, T., & He, C., et al. (2023). Variation in glucosinolates and the formation of functional degradation products in two brassica species during spontaneous fermentation. *Current Research in Food Science*, *6*, 100493. https://doi.org/https://doi.org/10.1016/j.crfs.2023.100493.
